# Case Report: Neuroretinitis versus hypertensive retinopathy secondary to Alport syndrome

**DOI:** 10.3389/fneur.2026.1745094

**Published:** 2026-01-22

**Authors:** Rohini Chahal, Amna M. Ali, Safa Ibrahim, Dina Abdelsalam, Andrew G. Lee

**Affiliations:** 1 The University of Texas Health Science Center at Houston, McGovern Medical School, Houston, TX, United States; 2 Blanton Eye Institute, Houston Methodist Hospital, Houston, TX, United States; 3 Departments of Ophthalmology, Neurology, and Neurosurgery, Weill Cornell Medicine, New York, NY, United States; 4 Department of Ophthalmology, University of Texas Medical Branch, Galveston, TX, United States; 5 Department of Ophthalmology, The University of Iowa Hospitals and Clinics, Iowa City, IA, United States

**Keywords:** acetazolamide, Alport syndrome, case report, hypertensive retinopathy, macular star, neuroretinitis

## Abstract

**Introduction:**

Alport syndrome (AS) is an inherited disease caused by the absence of type IV collagen from glomerular, cochlear, and ocular basement membranes. Ocular manifestations of AS include corneal opacity, peripheral and central fleck retinopathy, and temporal thinning on OCT. These findings in AS usually do not cause any vision loss. We report a case of bilateral vision loss with optic disc edema, macular edema, and macular star figure in AS.

**Case description:**

A 27-year-old woman presented with progressive, painless bilateral blurry vision over 2 weeks. She had genetically confirmed X-linked COL4A5 Alport syndrome and uncontrolled hypertension despite four antihypertensive medications. Visual acuity was 20/50 OU. Ishihara color testing was 6/14 OD and 2/14 OS. Fundoscopy showed moderate optic disc edema, a complete macular star, and tortuous vessel OU. Humphrey visual fields revealed severe constriction (mean deviation was −17.65 dB OS and −21.98 dB OD). Blood pressure was 163/93 mm Hg. OCT showed a peripapillary RNFL of 245 μm OD and 204 μm OS. MRI indicated an enlarged, partially empty sella and bilateral patulous optic nerve sheath. Lumbar puncture opening pressure was 13 cm H₂O with mildly elevated CSF protein and no pleocytosis. Infectious and inflammatory neuroretinitis workup was negative. She was started on acetazolamide 250 mg twice daily and transitioned to hemodialysis. After 9 months, vision improved to 20/30 OU, color plates to 10/14 OD and 8/14 OS, and OCT RNFL to 139 μm OD and 128 μm OS. Visual fields improved (mean deviation −3.13 dB OS, −3.8 dB OD).

**Conclusion:**

We consider the possibility of hypertensive retinopathy or idiopathic neuroretinitis in this patient with Alport syndrome. Given the disproportionate degree of macular edema and prominent macular star, in the absence of common manifestations of hypertensive retinopathy such as retinal hemorrhages or hard exudates, we suspect a possible superimposed neuroretinitis-like exudative mechanism driven by the absence of type IV collagen, providing structural support in Alport syndrome patients. This complex symptom presentation, to our knowledge, has never been described in the context of Alport syndrome. Further research is needed to better understand the relationship between Alport-related renal disease, hypertensive retinopathy, and neuroretinitis.

## Introduction

Alport syndrome (AS) is an inherited disease caused by the absence of type IV collagen from the glomerular, cochlear, and ocular basement membranes (1). AS mutations in COL4A3, COL4A4, or COL4A5 follow an X-linked inheritance pattern in 85% of cases. AS is characterized by a triad of nephritis, sensorineural hearing loss, and ocular abnormalities. The structural fragility of the renal basement membrane causes chronic kidney disease and is often the first indication of disease, initially manifesting as gross hematuria, edema, and systemic hypertension.

The ocular manifestations of AS usually occur in the cornea, lens capsule, and retina (2). Classic features include corneal opacity, peripheral and central fleck retinopathy, and temporal thinning on OCT (2, 3). These findings in AS usually do not cause vision loss. Systemic hypertension, however, can occur in AS and can present with secondary ocular manifestations of hypertensive retinopathy and small vessel ischemia (4, 5). We report a case of bilateral vision loss with optic disc edema, macular edema, and macular star figure in AS.

## Case description

A 27-year-old female presented with progressive, painless, bilateral blurry vision for over 2 weeks. Her past medical history was significant for a 4-year history of genetically confirmed X-linked COL4A5 AS. The patient’s mother and maternal grandfather had chronic kidney disease secondary to AS. She developed end-stage renal disease and erythropoietin deficiency anemia, began peritoneal dialysis, and has been on the kidney transplant list since diagnosis. Her blood pressure was uncontrolled despite being on four concurrent anti-hypertensive medications, with her systolic blood pressure often > 200 mm Hg and diastolic pressures up to 108 mm Hg. Her medications were carvedilol, clonidine, sacubitril/valsartan, and torsemide. One week prior to symptom onset, the patient had a recorded blood pressure of 180/120 mmHg, representing a marked increase from a measurement of 137/77 mmHg obtained 2 weeks earlier.

**Figure 1:**
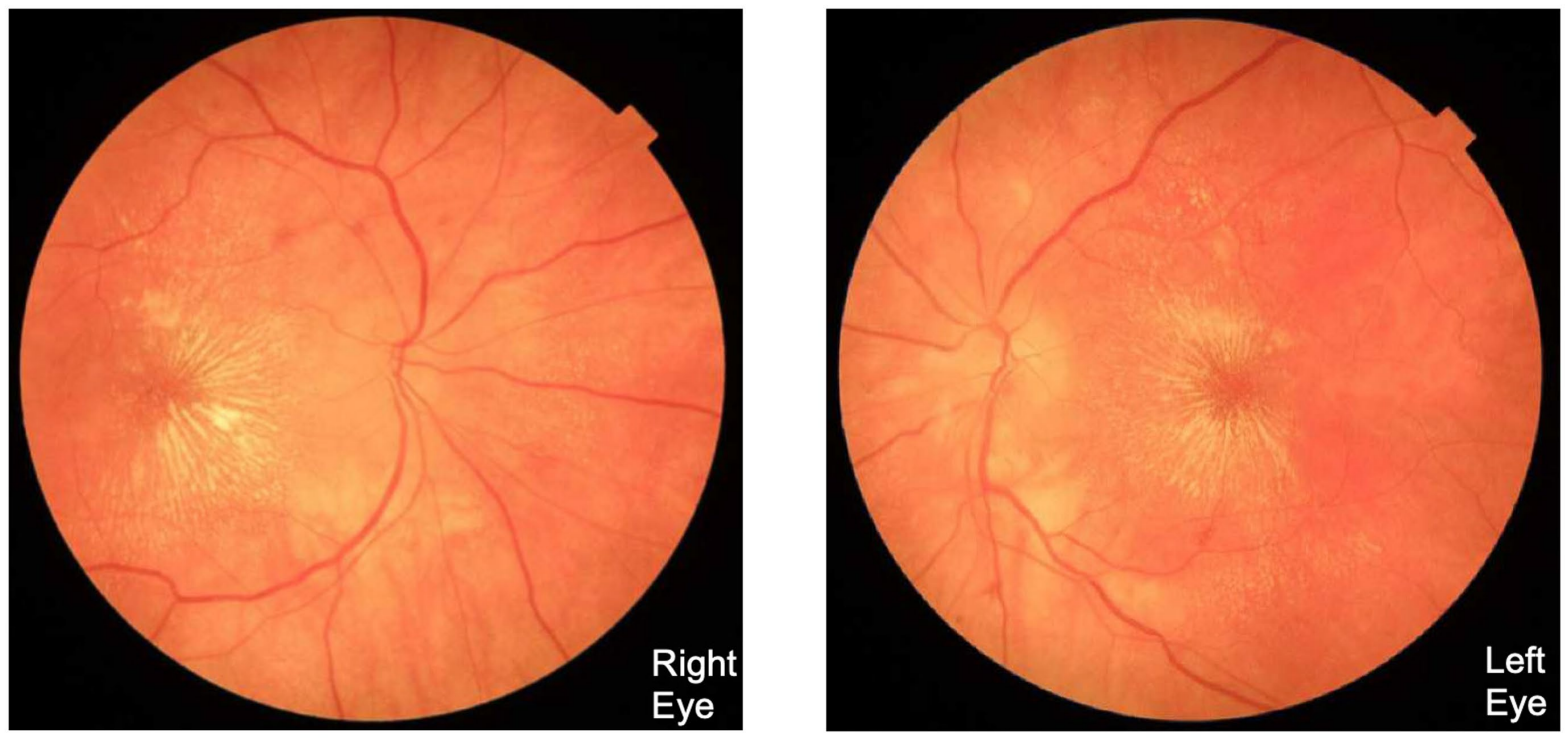
This fundus photograph, taken upon initial presentation at our hospital, demonstrates moderate optic disc edema, tortuous vessels, and macular star figure in both eyes.

On examination, the visual acuity was 20/50 in both eyes (OU). Ishihara color plates were correctly identified in 6/14 in the right eye (OD) and 2/14 in the left eye (OS). Ophthalmoscopy showed moderate optic disc edema, a complete macular star figure of exudate, and tortuous vessel OU (Figure 1). Automated perimetry (Humphrey visual field (HVF) 24-2) showed a mean deviation of −17.65 decibels (dB) OS and −21.98 dB OD, with predominantly central vision loss with diffuse constriction and superior and inferior arcuate defects OU. The remainder of the ocular exam was normal. The blood pressure measured 163/93 mm Hg.

**Figure 2:**
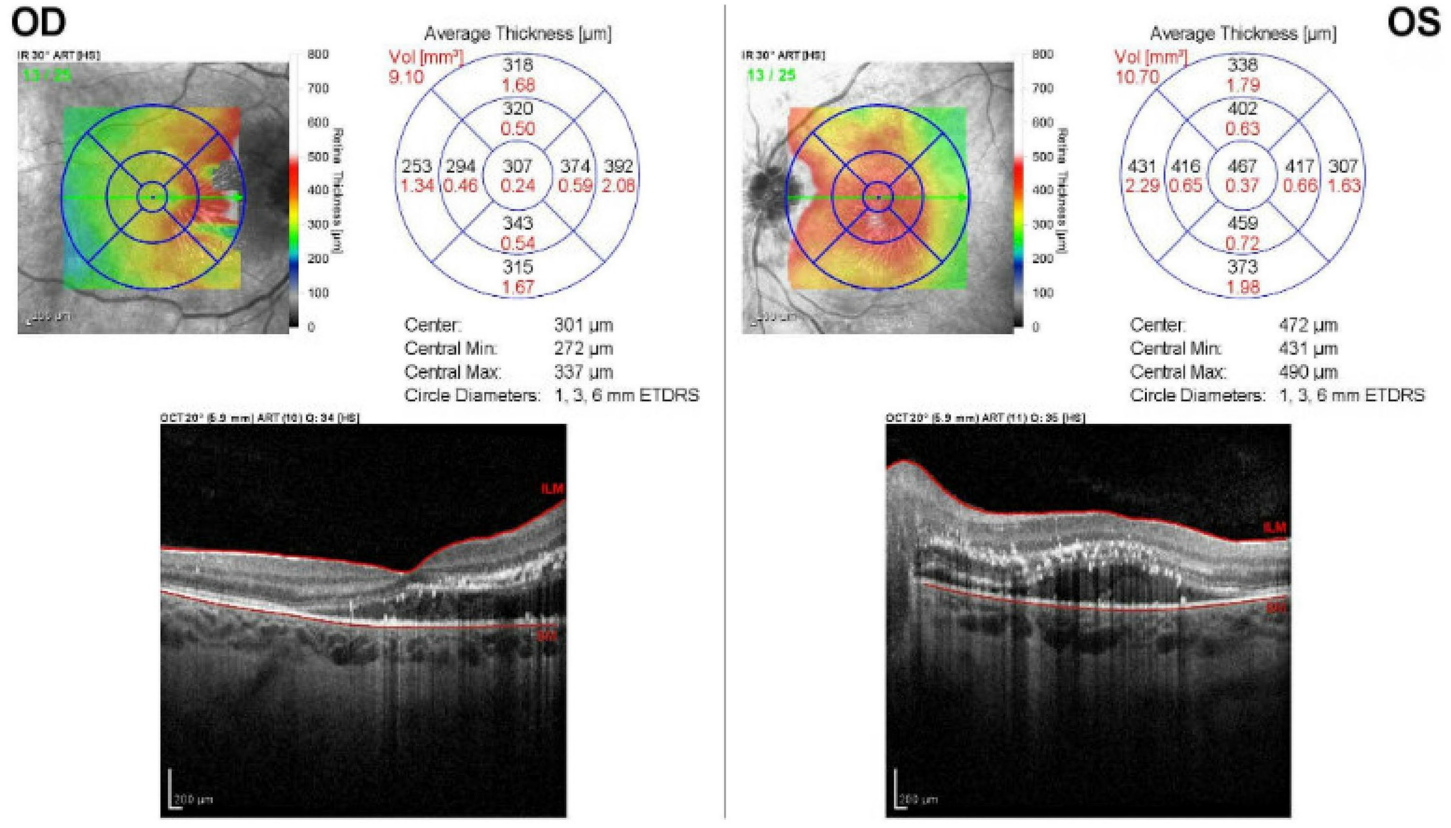
OCT macula on initial presentation shows bilateral intraretinal macular edema with prominent hyperreflective foci consistent with lipid exudates, more severe in the left eye, without significant subretinal fluid or cystoid change.

Optical coherence tomography (OCT) showed an optic nerve peripapillary retinal nerve fiber layer thickness (RNFL) of 245 microns OD and 204 microns OD. OCT macula demonstrated bilateral intraretinal macular edema with prominent hyperreflective foci consistent with lipid exudates, more severe in the left eye, without any significant subretinal fluid or cystoid change (Figure 2). Magnetic resonance imaging (MRI) indicated an enlarged, partially empty sella and bilateral patulous optic nerve sheath. MR Venogram demonstrated no venous sinus thrombosis. Serum testing for Bartonella, Lyme disease, tuberculosis (interferon gamma release assay), and syphilis was negative. A lumbar puncture showed opening pressure of 13 cm H_2_O. Cerebrospinal fluid (CSF) protein was mildly elevated, but no CSF pleocytosis was noted. She was started on 250 milligrams of acetazolamide twice a day and transitioned to hemodialysis after a nephrology consultation was obtained.

**Figure 3:**
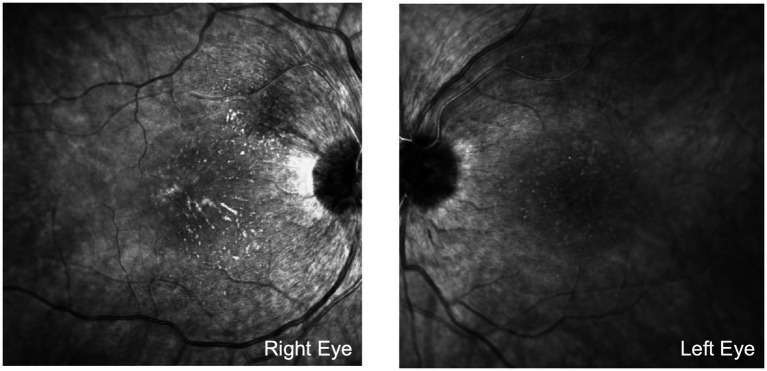
This fundus photograph was taken at the most recent follow-up visit, approximately 8 months after initial presentation at our hospital. Compared to Figure 1, the optic disc edema exhibits sharper disc margins, less hyperemia, and reduced peripapillary swelling, indicating improved optic nerve head edema. The macular star formation is more visible and organized, which indicates a delayed manifestation rather than worsening insult.

Two days later, the patient had a recorded serum sodium of 124, potassium of 6.1, and chloride of 90. Following normalization of serum electrolytes, acetazolamide was discontinued after 10 days of use. At follow-up examination 9 months later, the patient disclosed partial adherence to her hemodialysis schedule. The vision had improved to 20/30 OU, and Ishihara color plates improved to 10/14 OD and 8/14 OS. Ophthalmoscopy showed sharper disc margins, less hyperemia, and reduced peripapillary swelling, indicating improved optic nerve head edema (Figure 3). The macular star formation was more visible and organized, demonstrating a delayed manifestation rather than worsening insult. This aligned with improved findings of OCT RNFL of 139 microns OD and 128 microns OS. HVF testing revealed improvement in the visual field OU with a mean deviation of −3.13 dB OS and −3.8 OD dB.

## Discussion

Although AS is an X-linked disorder, the carrier state in females can manifest phenotypically. Previous studies have demonstrated that females with AS have various degrees of disease severity, with no genotype–phenotype correlation (6, 7). Lyonization is the process of X-chromosome inactivation, wherein one of the two X chromosomes in females is randomly silenced during embryonic development (3). This allows for the expression of X-linked recessive diseases in carrier state patients, and the unpredictable nature of the genotype–phenotype relationship.

This patient had ocular features of macular edema, eventually manifesting as a macular star figure. Macular edema is the pathological accumulation of fluid in the central retina, caused by the breakdown of the blood-retinal barrier (8). As leaked serum is resorbed, lipid exudates precipitate in a stellate pattern. When described in the same vignette as macular star figure, optic disc edema, and peripapillary RNFL thickening, the unifying etiology may be neuroretinitis (9).

Neuroretintis is defined as inflammation of the anterior optic nerve and peripapillary retina (10). This condition presents with a triad of vision loss, optic disc swelling, and macular star formation. The etiology can be organized as idiopathic and non-idiopathic causes, which include inflammatory and infectious diseases. In the case of our patient, typical testing for infectious (cat-scratch disease, lyme disease, syphillis, and tuberculosis) and inflammatory (sarcoidosis, systemic lupus erythematosus, and Behcet disease) etiologies was negative. However, 50% of neuroretinitis cases are idiopathic (11). This condition typically presents unilaterally, but bilateral cases have been previously described (12). Initial presentation is optic disc swelling, which may or may not have associated disc flame hemorrhages and intraretinal or subretinal fluid in the peripapillary region. The geography of vision loss is varied, with many patients complaining of central or ceco-central loss. This patient experienced bilateral central vision loss on Humphrey visual field testing.

Several other pathologies can lead to macular edema and macular star, including hypertensive retinopathy, caused by damaged blood vessels in the retina from high systemic blood pressures (13). Nephritis in AS can eventually lead to end-stage renal disease, as we saw in this patient. It is well documented that ESRD secondary to AS can cause systemic hypertension because of fluid overload (1). This hypertension can be further exacerbated by dialysis and can eventually manifest as hypertensive retinopathy in the eye (14, 15). This typically manifests with retinal hemorrhages, cotton wool spots, and macular edema in the affected eye.

On MRI, this patient displayed features suggestive of idiopathic intracranial hypertension (IIH) with an enlarged partially empty sella and bilateral patulous optic nerve sheath. However, the lumbar puncture opening pressure (OP) was 13 cm H_2_O. According to the Revised Diagnostic Criteria for the Pseudotumor Cerebri Syndrome and the 2018 IIH Consensus Guidelines, an OP of 6–25 cm H_2_O is considered the normal reference range (16, 17). Additionally, it is important to mention that the lumbar puncture positioning and sedation were not documented, and therefore, cerebrospinal fluid opening pressure interpretation is constrained and should not be used to infer raised intracranial pressure (ICP) in this case. It has been previously established that bilateral optic disc edema with a macular star formation in the setting of severe hypertension often lies on the spectrum of ischemic optic neuropathy rather than raised ICP (18). This would further support the hypothesis that the MRI findings are non-specific and do not override the normal OP.

It is important to differentiate the ocular findings of hypertensive retinopathy and neuroretinitis as they indicate separate etiologies and may require different treatments. The two ocular syndromes may be difficult to distinguish in this case, as there are several overlapping features, especially with a common macular star presentation (12–14, 19).

Table 1 demonstrates the common fundoscopic findings that can be used to diagnose patients with either ocular syndrome.

**Table 1:** Fundoscopic findings differentiating hypertensive retinopathy and neuroretinitis.

Exam findings	Hypertensive retinopathy	Neuroretinitis
Etiology	Elevated blood pressure damages retinal arterioles	Inflammatory or infectious optic neuropathy causing optic disc edema with secondary leakage into the outer retina
Optic disc	Optic disc edema is seen in malignant hypertension	Optic disc edema typically precedes macular star formation, affecting the macula secondarily
Macula	Macular star formation from lipid exudates in severe cases	Macular star formation from lipid exudates developing days to weeks following optic disc edema
Venous changes	Moderate venous dilation due to retinal ischemia	Minimal/mild venous congestion; not a defining feature
Retinal arterioles	Narrowing; AV nicking	Typically normal caliber
Hemorrhages/Exudates	Flame-shaped hemorrhage, cotton-wool spots, hard exudates	Minimal hemorrhage, cotton-wool spots uncommon, macular exudates within the outer plexiform layer
Laterality	Usually bilateral and symmetric; may be asymmetric	Usually unilateral; bilateral involvement is uncommon but reported

Some patients may present with symptoms shared between the two conditions, and physical exam findings that may indicate involvement of both hypertensive retinopathy and neuroretinitis. It is difficult to determine if they are related or coincidental findings in such patients. This case described a patient with findings that supported a diagnosis of hypertensive retinopathy, given her long history of uncontrolled hypertension and end-stage renal disease, and the fundoscopic findings of a bilateral macular star figure, retinal arteriolar narrowing, and cotton wool spots. However, typical features of hypertensive retinopathy, such as hard exudates and retinal hemorrhages, are missing. The blood pressure was 163/93 mmHg upon presentation at our institution, which does not meet malignant hypertension criteria (>200/130 mmHg with evidence of acute microvascular changes affecting various organs), but does meet criteria for resistant hypertension as the patient was on four anti-hypertensives (20, 21). On the other hand, this patient also had findings that supported a diagnosis of neuroretinitis, given the presence of the typical triad – vision loss, optic disc swelling, and macular star formation – along with findings of peripapillary RNFL thickening.

Neuroretinitis represents a phenotypic pattern defined by optic disc edema with secondary lipid exudation in a macular star configuration rather than a single etiologic process (12). In this patient, the presence of bilateral optic disc edema, peripapillary RNFL thickening, cotton wool spots, and arteriolar narrowing in the setting of severe resistant hypertension is most consistent with hypertensive retinopathy as the primary driver. However, the disproportionate degree of macular edema and prominent macular star, in the absence of widespread retinal hemorrhages or hard exudates, suggests a possible superimposed neuroretinitis-like exudative mechanism characterized by capillary permeability rather than diffuse retinal ischemia.

In the eye, type IV collagen contributes to the integrity of the vascular basement membranes and the inner blood-retinal barrier (22). It is well documented that when the blood-retinal barrier breaks down, excess fluid accumulates, resulting in macular edema. This is the established pathophysiology for diabetic retinopathy, retinopathy of prematurity, ocular inflammatory diseases, among other ocular disorders. Defects in this collagen network, such as those seen in Alport syndrome, may lower the threshold for inner blood–retinal barrier disruption and cause retinal capillaries to become more permeable and mechanically fragile. Under conditions such as hypertensive stress, this may amplify leakage of plasma constituents.

We propose that this patient’s presentation reflects overlapping mechanisms, in which hypertensive retinopathy produces bilateral ischemic disc edema while concomitant structural microvascular vulnerability facilitates a secondary neuroretinitis-like exudative response, rather than two independent disease entities.

Several considerations needed to be made for the treatment of our patient. Hypertensive retinopathy is treated with lifestyle changes and systemic blood pressure control with anti-hypertensive medications (19). In patients with ESRD, hemodialysis can play a critical role by enabling aggressive volume management and facilitating blood pressure control in cases of refractory or malignant hypertension (23). Idiopathic neuroretinitis, on the other hand, typically resolves spontaneously without treatment (10). If the neuroretinitis was a secondary effect of hypertensive retinopathy, this could also resolve with blood pressure control. Given the treatment-resistant hypertension and concerning patient symptoms, the patient was also given acetazolamide, a carbonic anhydrase inhibitor that is eliminated by the renal system and potentially toxic to patients with severe kidney disease, and was started on hemodialysis per the recommendation by nephrology (24). During treatment with acetazolamide, this patient experienced electrolyte abnormalities. The recorded sodium was 122, potassium was 6.1, and chloride was 90. The patient reported partial adherence with her hemodialysis schedule, and her electrolyte values normalized following closer adherence to hemodialysis. We believe adherence to dialysis is critical in ESRD patients undergoing acetazolamide treatment to avoid electrolyte abnormalities. Acetazolamide has also been shown to lower systemic blood pressure. Although the patient’s vision improved, it is difficult to say exactly how it improved. The acetazolamide treatment was confounded by the simultaneous transition to hemodialysis. Thus, the patient’s clinical response can be attributed to the patient’s response to this multimodal treatment strategy targeting the underlying renal driver through hemodialysis with adjunctive acetazolamide.

In conclusion, we consider the possibility of hypertensive retinopathy or idiopathic neuroretinitis as causes of the bilateral optic disc edema, macular edema, macular star formation, peripapillary RNFL thickening, and vessel tortuosity seen in this patient with Alport syndrome. It is compelling to believe that our patient’s kidney disease may have caused systemic hypertension and associated hypertensive retinopathy findings. However, the disproportionate degree of macular edema and prominent macular star, in the absence of common manifestations of hypertensive retinopathy such as retinal hemorrhages or hard exudates, suggest a possible superimposed neuroretinitis-like exudative mechanism characterized by capillary permeability rather than diffuse retinal ischemia, and driven by the absence of type IV collagen in Alport syndrome patients. This complex symptom presentation, to our knowledge, has never been described in the context of Alport syndrome. Further research is needed to better understand the relationship between Alport-related renal disease, hypertensive retinopathy, and neuroretinitis.

## Data Availability

The original contributions presented in the study are included in the article/Supplementary material, further inquiries can be directed to the corresponding author.
