# Supplementary figures and images for: Case Report: Neuroretinitis versus hypertensive retinopathy secondary to Alport syndrome
Source: Front Neurol. 2026 Jan 22;17:1745094. doi: 10.3389/fneur.2026.1745094 (PMC12873304; doi:10.3389/fneur.2026.1745094)

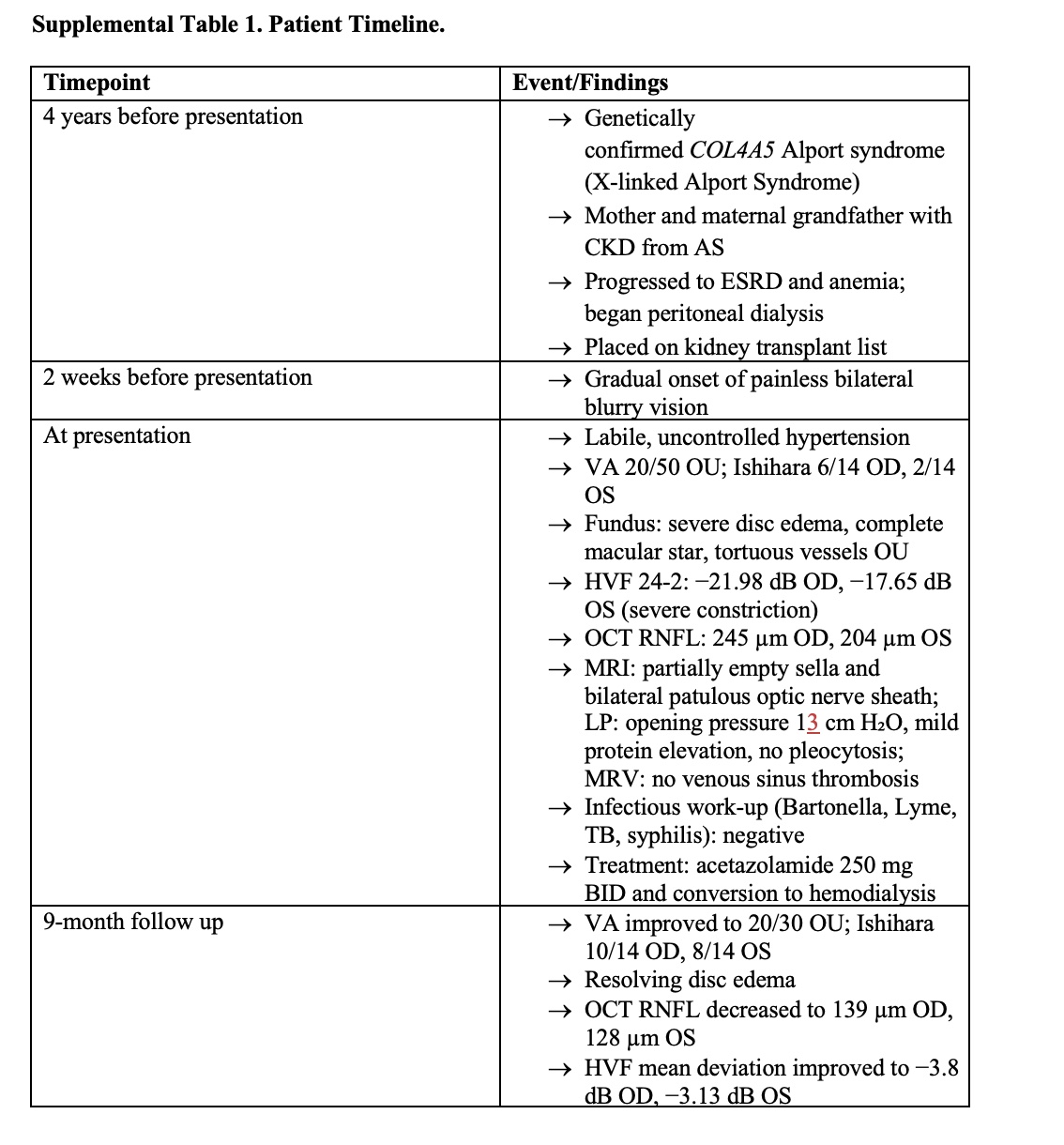

Supplement: Supplementary file 1 [file Image_1.JPEG]
